# Case Report: Liver Cysts and SARS-CoV-2: No Evidence of Virus in Cystic Fluid

**DOI:** 10.3389/fsurg.2021.677889

**Published:** 2021-06-18

**Authors:** Francesco Enrico D'Amico, Dajana Glavas, Giulia Noaro, Domenico Bassi, Riccardo Boetto, Enrico Gringeri, Maurizio De Luca, Umberto Cillo

**Affiliations:** ^1^Department of Surgical, Oncological and Gastroenterologica Sciences (DiSCOG), University of Padova, Padua, Italy; ^2^Hepatobiliary and Liver Transplant Surgery, Padova Teaching Hospital, Padua, Italy; ^3^Department of Surgery, Montebelluna Hospital ULSS2, Montebelluna, Italy

**Keywords:** liver cyst infection, liver benign disease, CoV-2, jaundice, fenestration cyst

## Abstract

**Background:** In December 2019, an outbreak of pneumonia, caused by a new type of coronavirus, named severe acute respiratory syndrome coronavirus 2 (SARS-CoV-2). It quickly spread worldwide, resulting in a pandemic. The clinical manifestations of SARS-CoV-2 range from mild non-specific symptoms to severe pneumonia with organ function damage. In addition, up to 60% of patients have liver impairment or dysfunction, confirmed by several studies by the presence of SARS-CoV-2 in the liver tissue.

**Methods:** We report two cases of symptomatic liver cyst requiring fenestration after recent SARS-CoV-2 infection. Both patients had hospital admission due to documented SARS-CoV-2 infection. Recently, after the infection, they developed symptoms caused by an enlarged hepatic cyst: one had abdominal pain, and the other had jaundice. They underwent surgery after two negative swab tests for SARS-CoV-2.

**Results:** Cystic fluid was sent for microbiological test, and real-time fluorescence polymerase chain reaction COVID-19 nucleic-acid assay of the cyst fluid was found to be negative in both cases.

**Discussion:** Although there are no current data that can document a viral contamination of cystic fluid, there are data that document a hepatotropism of COVID-19 virus. Herein we report that after viral clearance at pharyngeal and nasal swab, there is no evidence of viral load in such potential viral reservoir.

## Background

In December 2019, an outbreak of pneumonia, caused by a new type of coronavirus, named as severe acute respiratory syndrome coronavirus 2 (SARS-CoV-2), emerged in China. It quickly spread worldwide, resulting in a pandemic.

SARS-CoV-2 has been responsible for more than 140,332,386 confirmed cases, with more than 3 million deaths around the world, as recently reported by the World Health Organization (1).

The clinical manifestations of coronavirus disease 2019 (COVID-19) range from mild non-specific symptoms, to severe pneumonia with damage to organ function. Common symptoms are ageusia, anosmia, fever, cough, fatigue, dyspnea, myalgia, and headache; less common symptoms are sore throat, rhinorrhea, chest pain, hemoptysis, conjunctival congestion, diarrhea, nausea, vomiting, and other gastrointestinal symptoms (2, 3).

SARS-CoV-2 hepatotropism is a quite controversial topic in literature as a direct evidence of specific liver tropism is challenging to obtain and yet not fully documented (4–6). From 43% up to 60% of patients have elevation of liver enzymes and liver impairment. This is confirmed by several studies, which conclude that there is a potential presence of SARS-CoV-2 in the liver tissue (7–13). Furthermore, patients with severe infection by SARS-CoV-2 seem to have higher rates of liver dysfunction. It is currently uncertain whether the COVID-19–related liver injury is due to the direct viral infection of liver parenchyma and/or to coexisting conditions, such as the use of hepatotoxic drugs, the systemic inflammatory response, and multiple organ dysfunction caused by SARS-CoV-2 infection (14–22). As a viral hepatotropism has been postulated, to date, it is unclear if the virus can be present, after pulmonary and systemic clearance, in retained body fluids such as liver cystic fluid. Herein, we discuss two cases of symptomatic liver cysts, surgically treated, in patients with recently confirmed SARS-CoV-2 infection. The aim of this study is to verify the persistence or absence of viral load of COVID-19 virus in the cystic fluid after negative pharyngeal swab tests.

## Case Presentation

### Patient 1

On May 20, 2020, a 60-year-old woman came to the emergency room of Padova Teaching Hospital, with a 2-day history of high-intensity abdominal pain, diarrhea, and anemia (hemoglobin level at 8.1 g/dL), without any sign or symptom of gastrointestinal bleeding. She was afebrile, and she denied fever, cough, and shortness of breath in the previous days.

The patient had a medical history of extramammary Paget disease, right lower lung lobectomy for pulmonary adenocarcinoma in 2018, and an asymptomatic liver cyst on regular follow-up.

**Figure 1:**
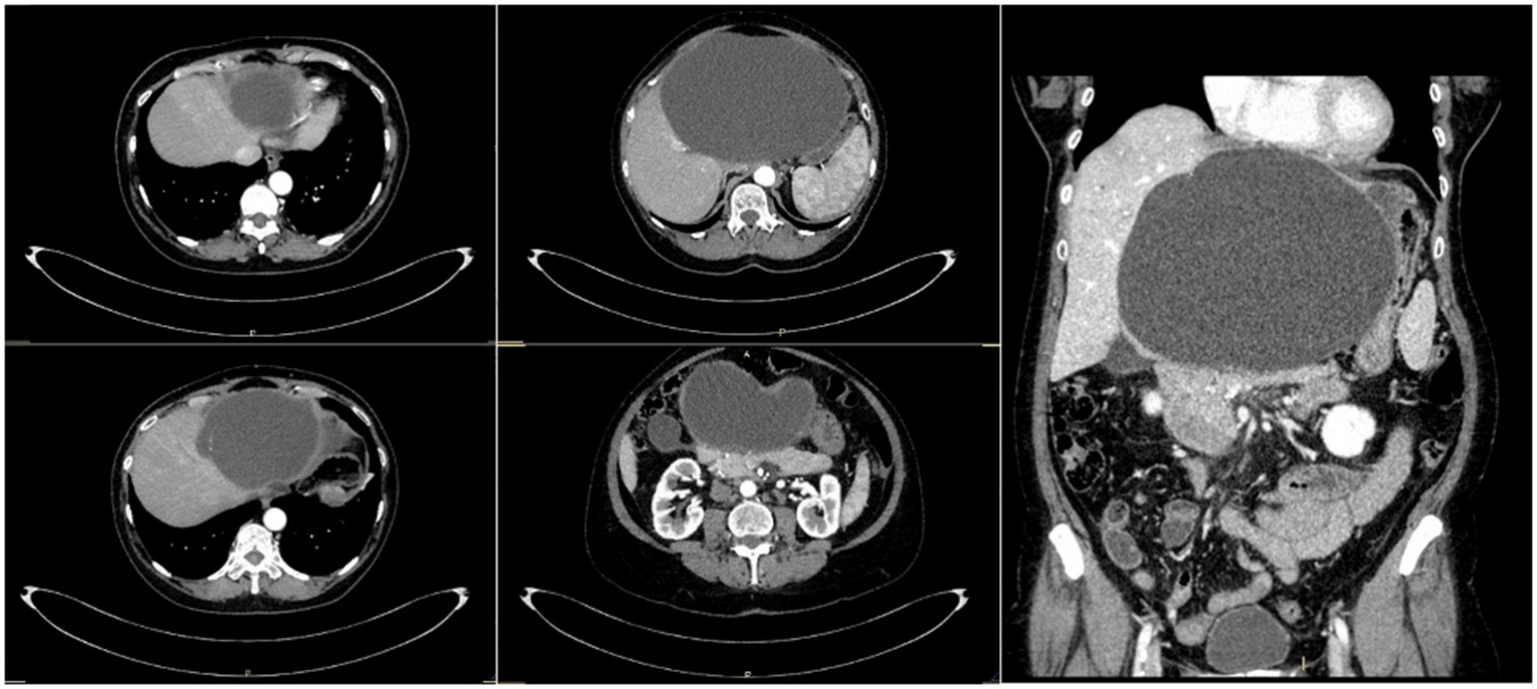
CT scan of Patient 1 showing the liver cyst that occupies all the left hemi-liver.

At admission, an abdominal computed tomography (CT) scan revealed a 20 × 13.6-cm simple hepatic cyst, which caused compression on the gallbladder, stomach, and pancreas and Wirsung dilatation (Figure 1). No sign of active bleeding was detected at the CT scan, and the cyst appeared enlarged, when compared with previous follow-up radiological examinations.

At admission, a real-time fluorescence polymerase chain reaction (RT-PCR) assay of pharyngeal swabs was performed, and the patient tested positive for COVID-19 nucleic acid. Blood tests showed a normal white blood cell (WBC) count (5.26 × 10^9^/L, reference range = 4.40–11.00 × 10^9^/L), elevated blood levels for C-reactive protein (CRP) (11 mg/L, reference range = 0–6 mg/L), and normal levels for procalcitonin (0.06 μg/L, reference range = 0.00–0.50 μg/L). Three consecutive samples of fecal occult blood test resulted negative.

The patient was treated with hydroxychloroquine and azithromycin, according to regional guidelines in force at that time. Because of the symptoms caused by the liver cyst, after healing from the SARS-CoV-2 infection, and with two consecutive negative swab tests (6 and 7 days after the first positive swab), the patient was transferred to our facility for liver surgery.

At admission to the surgical department on May 29, 2020, the patient had normal body temperature at 35.7°C. Three consecutive RT-PCR assays of pharyngeal swabs and of sputum tested negative for SARS-CoV-2 nucleic acid. Blood tests showed a normal WBC count of 4.41 × 10^9^/L; the alanine aminotransferase (ALT), aspartate aminotransferase (AST), and total bilirubin levels were 14 U/L (reference range = 7–35 U/L), 22 U/L (reference range = 10–35 U/L), and 10.8 μmol/L (reference range = 1.7–17.0 μmol/L), respectively. Anti-*Echinococcus* antibodies were also performed, and they tested negative.

**Figure 2:**
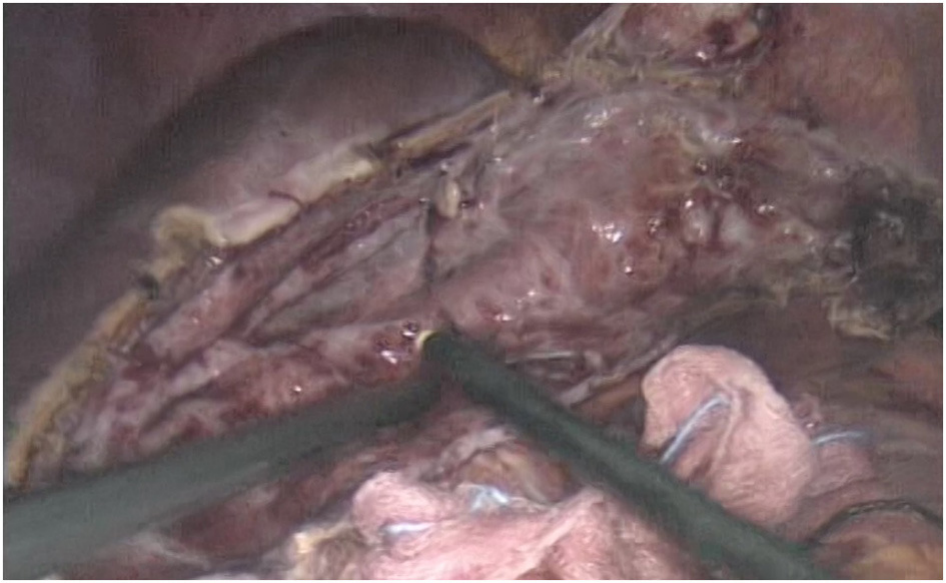
Patient 1 Intraoperative image after completing the cyst fenestration.

As planned, the patient underwent a laparoscopic cyst fenestration, the day after admission (Figure 2). The video laparoscopic exploration and the intraoperative ultrasound documented the already known per-magna hepatic cyst, with complete atrophy of the left hepatic lobe. After cyst incision, 1,500 mL of transparent “rock water” fluid was drained, and the cyst fenestration was performed. The cystic wall was sent for pathology, and the cystic fluid for cytology: the first was consistent with simple biliary cist, the latter with granulocytes, lymphocytes, and macrophages. Cystic fluid tested negative at microbiological research. In particular, the RT-PCR COVID-19 nucleic-acid assay of the cystic fluid was found to be negative. The patient was discharged from the hospital on the second post-operative day with no complications.

### Patient 2

On March 27, 2020, an 80-year-old man was admitted to a Local Hospital Medical Department for asthenia, headache, and general malaise. He denied fever and dyspnea. The patient had a medical history of benign prostatic hypertrophy, chronic cerebral vasculopathy, dyslipidemia, and a simple liver cyst of 35 mm on regular follow-up. At admission, oxygen saturation was ~89%, blood tests showed normal WBC count (4.49 × 10^9^/L, reference range = 3.60–10.50 × 10^9^/L), normal blood level of procalcitonin (<0.05 ng/mL, reference range = 0.0–0.5 ng/mL), but elevated blood levels of CRP (5.6 mg/dL, reference range = 0.00–0.50 mg/dL). Liver function tests were normal: AST 28 U/L (reference range = 0–50 U/L), ALT 30 U/L (reference range = 0–50 U/L), γ-glutamyl transferase (GGT) 171 U/L (reference range = 0–55 U/L), total bilirubin 0.6 mg/dL (reference range = 0.2–1.2 mg/dL), and direct bilirubin 0.2 mg/dL (reference range = 0.0–0.2 mg/dL). The RT-PCR assay of pharyngeal-nose swab tested positive for COVID-19 nucleic acid. The chest x-ray showed a bilateral pneumonia. Given the rapid clinical deterioration, positive-pressure ventilation with a continuous positive airway pressure was undertaken. The patient was initially treated with hydroxychloroquine and azithromycin, and subsequently with darunavir and ritonavir, according to the regional guidelines. After 7 days, the patient was weaned from high-flow ventilatory support, and he was discharged home 2 weeks after admission, continuing home surveillance until 2 consecutive swab tests were negative for COVID-19 nucleic acid.

On June 27, 2020, the patient was admitted to the same Local Hospital Medical Department for jaundice, weakness, general discomfort, bloated abdomen, and diarrhea. He was afebrile, with normal vital signs. He denied recent respiratory symptoms. At the time of the admission, the RT-PCR COVID-19 nucleic-acid assay of pharyngeal swab was negative. Blood tests showed normal WBC count (10.43 × 10^9^/L), a slight increase in CRP level (2.28 mg/dL), and important alterations of liver function tests, with a total bilirubin of 16.6 mg/dL, direct bilirubin of 9.2 mg/dL, AST 477 U/L, ALT 614 U/L, and GGT 1347 U/L.

**Figure 3:**
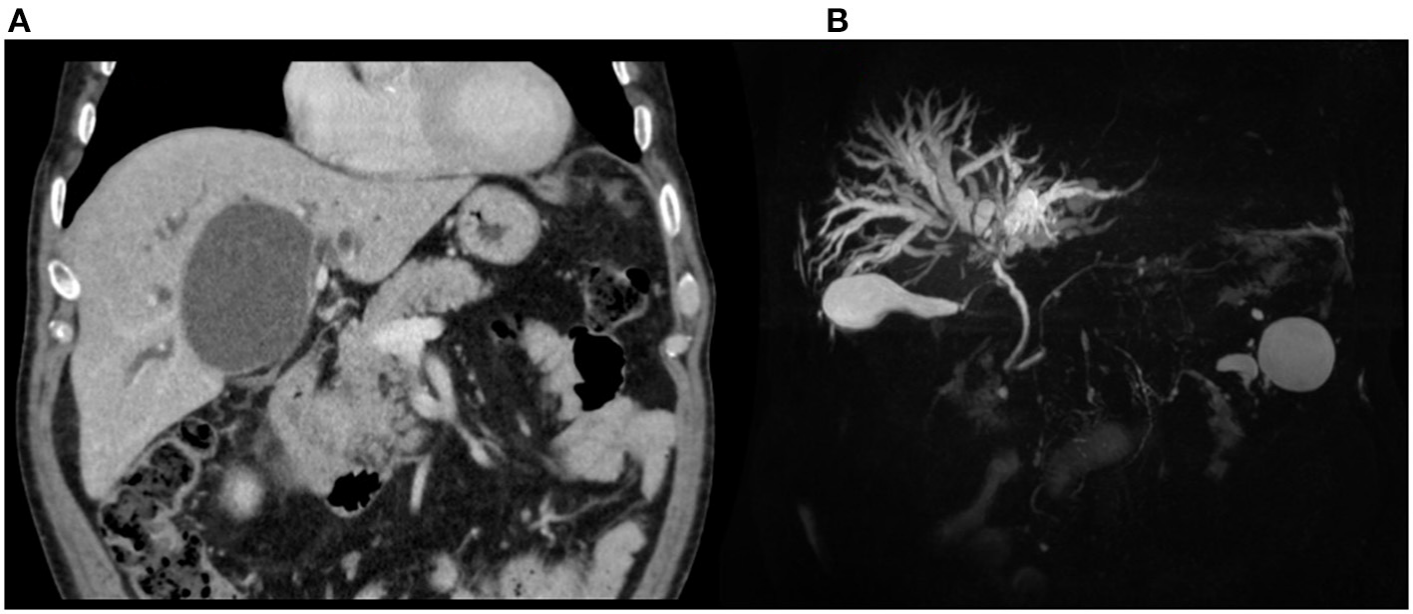
Patient 2, preoperative imaging: **(A)** CT scan and **(B)** MRCP demonstrating biliary confluence compression with dilatation.

An abdominal CT scan was performed, revealing an enlargement of the hepatic cyst to 9 × 7.5 cm in segment 4b (Figure 3A). The magnetic resonance cholangiopancreatography described a compression of the main biliary confluence, with dilatation of the intrahepatic biliary tracts, due to the enlargement of the liver cyst (Figure 3B). The cyst had no signs of active bleeding or communication with the biliary tract. Blood research of anti-*Echinococcus* antibodies tested negative. After a multidisciplinary discussion, surgical intervention was recommended, and the patient was transferred to the surgical department. The patient underwent a laparoscopic cyst fenestration: 500 mL of clear liquid was drained and sent for microbiological research and cytology, whereas the cystic wall was sent for pathology. The pathological analysis was consistent with normal hepatic parenchyma with no alterations, and the cytology was negative for neoplastic cells. Cystic fluid was found to be negative for both microbiological and RT-PCR SARS-CoV-2 nucleic acid research. Post-operative blood tests showed a progressive reduction of hyperbilirubinemia: on day 4, total bilirubin dropped to 5.6 mg/dL, and direct bilirubin to 2.5 mg/dL. AST, ALT, and GGT decreased to 119, 271, and 329 U/L, respectively. The patient was discharged in good clinical condition without surgical complications on the fifth post-operative day.

## Discussion and Conclusions

Liver impairment has been reported as a frequent manifestation of SARS-CoV-2 pandemic (23). The mechanism remains still unclear and controversial, as liver injury could be the result of the direct viral infection, as well as a reflection of the severe inflammatory response, hypoxic hepatitis as a result of anoxia, and it could be also caused by the use of hepatotoxic drugs. Nevertheless, several studies confirm the ability of SARS-CoV-2 to directly affect liver tissue, by binding angiotensin-converting enzyme 2 (ACE2) receptors. *In vitro* studies identified ACE2 cell surface receptor expressed in cholangiocytes as the host receptor for viral entry, leading to cholangiocyte dysfunction and liver injury (23–26). Moreover, pathological studies confirmed the presence of the virus in liver tissue (9, 24, 27–29). However, liver tropism remains a debated topic in literature (Table 1). This is due to the lack of solid research models and paucity of histological studies due to difficulty of obtaining samples of liver tissue during and after SARS-CoV-2 infection. Currently, the most solid data came from post-mortem or *in vitro* studies on liver organoids or human and animal cell lines (10–12, 14, 19, 20, 30).

**Table 1:** Studies on hepatotropism available in the literature.

**References**	**Number of patients**	**Type of study: *in vitro*/*in vivo*/post-mortem**	**Liver tropism**	**SARS-CoV-2 presence**
Tian et al. (28)	4	Post-mortem biopsies	Yes	SARS-CoV-2 RNA isolated from liver tissue in 1 of 4 patients
Chai et al. (26)	4	Post-mortem tissue sample	Yes	
Chu et al. (9)	9	Human cell lines	Yes	
Yang et al. (10)		Adult liver hepatocyte and cholangiocytes organoids	Yes	
Zhao et al. (19)	2	Human liver ductal organoids	Yes	
Wang et al. (12)	2	Post-mortem	Yes	Viral particles were observed in the cytoplasm of hepatocytes in two cases
Fassan et al. (30)	3 + 25	3 *in vivo* liver biopsies; 25 post-mortem tissue samples	Not specific	No viral particles detected
De Smet et al. (5)	3	Human liver datasets	No (technical limitations reported)	
Sonzogni et al. (11)	30 + 18	Post-mortem	NO (Vascular related damage)	SARS-CoV-2 detected in 15/22 samples inside blood cloths or endothelial cells cytoplasm
Chau et al. (14)	3	*In vivo* liver biopsy	Yes	SARS-CoV-2 RT-PCR positive in all liver biopsies but not in the serum
Pirola et al. (20)		*In vitro*	Yes	
Schurink et al. (29)	11	Post-mortem	Yes	SARS-CoV2 viral presence detected in 2/11 patients

Hepatic cysts are fluid-filled cavities located in the liver. Prevalence is estimated to be between 2.5 and 18.0%, of the general population. Simple cysts tend to occur more commonly in the right lobe, and they are more prevalent in women. The female-to-male ratio is ~1.5:1. In most cases, simple cysts are asymptomatic and incidentally discovered by ultrasound or CT scan, and they have a benign course. Large cysts are more likely to be symptomatic, causing abdominal pain, discomfort, compression of the biliary tree, or leading to other complications such as spontaneous rupture bleeding and infection of cystic fluid (31). Several therapeutic approaches have been described for symptomatic, large, simple cysts. Wide unroofing and cyst resection have been associated with a relatively low incidence of cyst recurrence and post-operative complications (32).

In the two cases herein described, both patients had symptoms caused by an increase in size of their liver cyst. The first patient had a history of dyspepsia, abdominal pain, and CT scan evidence of gallbladder, stomach, and pancreas compression, with pancreatic duct dilatation. The second patient presented with jaundice secondary to the compression of the biliary bifurcation.

As the ability of SARS-CoV-2 to directly bind to ACE2-positive cholangiocytes has been documented, as well as liver tissue damage, we wondered if viral load could be detected in fluids, such as hepatic cystic fluid, even after SARS-CoV-2 pulmonary clearance. Indeed, to our knowledge, there has not been a study to report the presence of RT-PCR nucleic acid of COVID-19 in cystic fluid, to date.

In both cases herein presented, patients underwent surgery after recovery from documented SARS-CoV-2, and the RT-PCR nucleic acid of COVID-19 on the cystic fluid, collected during the operations, tested negative. Moreover, pathological reports did not document any viral presence on the cystic walls. Interestingly, both patients presented with symptoms caused by an increase in cyst size, after SARS-CoV-2 infection. We do not know if the recent systemic inflammatory response to COVID-19 could have played a role in this acute cyst enlargement.

In conclusion, we report the clinical course of two patients (one male and one female) with a recent documented history of SARS-CoV-2 infection, and with symptomatic hepatic cyst, who underwent surgery after clearance of the viral load, confirmed on pharyngeal swab. Although there are no current data that can document a viral contamination of cystic fluids, we can report that after viral clearance at pharyngeal and nasal swab, there is no evidence of viral load in such a potential viral reservoir as the cystic fluid. Considering the novelty of SARS-CoV-2 disease and the poor understanding of it, we can expect more data in the future to improve our knowledge of the biology and its already proven hepatotropism. Despite the limitations of this article, as we could not provide any evidence of viral presence in cyst fluid or in the hepatic cells during SARS-CoV-2 infection, we think that this information can be helpful for the surgical planning of those patients with hepatic cysts and previous SARS-CoV-2 disease, who need liver surgery (such as liver resection for malignant or benign lesions, or fenestration of symptomatic liver cysts). We can speculate that there is no need for extra caution for operators during surgery. In addition, as the number of infected people is constantly increasing, this report could be useful also to discuss the safety of liver transplantation from donors with known previous COVID 19 infection, good hepatic function, and the presence of a liver cyst. Further studies are necessary to assess the extent and the persistence of liver damage in COVID-19 patients.

## Data Availability Statement

The raw data supporting the conclusions of this article will be made available by the authors, without undue reservation.

## Ethics Statement

Ethical review and approval was not required for the study on human participants in accordance with the local legislation and institutional requirements. The patients/participants provided their written informed consent to participate in this study. Written informed consent was obtained from the individual(s) for the publication of any potentially identifiable images or data included in this article.

## Author Contributions

FD'A idea, draft writing, and surgical procedure. DG draft writing and specimen collection. GN review, surgical procedure, and specimen collection. DB, RB, and EG: review. MD: surgical procedure. UC: review, surgical procedure, and idea. All authors contributed to the article and approved the submitted version.

## Conflict of Interest

The authors declare that the research was conducted in the absence of any commercial or financial relationships that could be construed as a potential conflict of interest.

## Abbreviations

SARS-COV-2: severe acute respiratory syndrome coronavirus 2
COVID-19: coronavirus disease 2019
CT scan: computed tomography scan
RT-PCR: real-time fluorescence polymerase chain reaction
ALT: alanine aminotransferase
AST: aspartate aminotransferase
WBC: white blood cell
CRP: C-reactive protein
GGT: γ-glutamyl transferase
ACE2: angiotensin-converting enzyme 2.

## References

[1] WHO. WHO Coronavirus Disease (COVID-2019) Situation Reports. Available online at: https://www.who.int/emergencies/diseases/novel-coronavirus-2019/situation-reports/

[2] Ahn DG Shin HJ Kim MH Lee S Kim HS Myoung J. Current status of epidemiology, diagnosis, therapeutics, and vaccines for novel coronavirus disease 2019 (COVID-19). J Microbiol Biotechnol. (2020) 30:313–24. 10.4014/jmb.2003.03011 32238757 PMC9728410

[3] Ge H Wang X Yuan X Xiao G Wang C Deng T. The epidemiology and clinical information about COVID-19. Eur J Clin Microbiol Infect Dis. (2020) 39:1011–9. 10.1007/s10096-020-03874-z 32291542 PMC7154215

[4] Marjot T Webb GJ Barritt ASt Moon AM Stamataki Z Wong VW. COVID-19 and liver disease: mechanistic and clinical perspectives. Nat Rev Gastroenterol Hepatol. (2021) 18:348–64. 10.1038/s41575-021-00426-4 33692570 PMC7945972

[5] De Smet V Verhulst S van Grunsven LA. Single cell RNA sequencing analysis did not predict hepatocyte infection by SARS-CoV-2. J Hepatol. (2020) 73:993–5. 10.1016/j.jhep.2020.05.030 32473193 PMC7253986

[6] Wiśniewska H Skonieczna-Zydecka K Parczewski M Niścigorska-Olsen J Karpińska E Hornung M. Hepatotropic properties of SARS-CoV-2-preliminary results of cross-sectional observational study from the first wave COVID-19 pandemic. J Clin Med. (2021) 10:672. 10.3390/jcm10040672 33572429 PMC7916209

[7] Mao R Qiu Y He JS Tan JY Li XH Liang J. Manifestations and prognosis of gastrointestinal and liver involvement in patients with COVID-19: a systematic review and meta-analysis. Lancet Gastroenterol Hepatol. (2020) 5:667–78. 10.1016/S2468-1253(20)30126-6 32405603 PMC7217643

[8] Xu Z Shi L Wang Y Zhang J Huang L Zhang C. Pathological findings of COVID-19 associated with acute respiratory distress syndrome. Lancet Respir Med. (2020) 8:420–2. 10.1016/S2213-2600(20)30076-X 32085846 PMC7164771

[9] Chu H Chan JF Yuen TT Shuai H Yuan S Wang Y. Comparative tropism, replication kinetics, and cell damage profiling of SARS-CoV-2 and SARS-CoV with implications for clinical manifestations, transmissibility, and laboratory studies of COVID-19: an observational study. Lancet Microbe. (2020) 1:e14–23. 10.1016/S2666-5247(20)30004-5 32835326 PMC7173822

[10] Yang L Han Y Nilsson-Payant BE Gupta V Wang P Duan X. A human pluripotent stem cell-based platform to study SARS-CoV-2 tropism and model virus infection in human cells and organoids. Cell Stem Cell. (2020) 27:125–36.e7. 10.1016/j.stem.2020.06.015 32579880 PMC7303620

[11] Sonzogni A Previtali G Seghezzi M Grazia Alessio M Gianatti A Licini L. Liver histopathology in severe COVID 19 respiratory failure is suggestive of vascular alterations. Liver Int. (2020) 40:2110–6. 10.1111/liv.14601 32654359 PMC7404964

[12] Wang Y Liu S Liu H Li W Lin F Jiang L. SARS-CoV-2 infection of the liver directly contributes to hepatic impairment in patients with COVID-19. J Hepatol. (2020) 73:807–16. 10.1016/j.jhep.2020.05.002 32437830 PMC7211738

[13] Ali N. Relationship between COVID-19 infection and liver injury: a review of recent data. Front Med. (2020) 7:458. 10.3389/fmed.2020.00458 32793619 PMC7385135

[14] Chau TN Lee KC Yao H Tsang TY Chow TC Yeung YC. SARS-associated viral hepatitis caused by a novel coronavirus: report of three cases. Hepatology. (2004) 39:302–10. 10.1002/hep.20111 14767982 PMC7165792

[15] Feng G Zheng KI Yan QQ Rios RS Targher G Byrne CD. COVID-19 and liver dysfunction: current insights and emergent therapeutic strategies. J Clin Transl Hepatol. (2020) 8:18–24. 10.14218/JCTH.2020.00018 32274342 PMC7132016

[16] Lee IC Huo TI Huang YH. Gastrointestinal and liver manifestations in patients with COVID-19. J Chin Med Assoc. (2020) 83:521–3. 10.1097/JCMA.0000000000000319 32243269 PMC7176263

[17] Wang H Qiu P Liu J Wang F Zhao Q. The liver injury and gastrointestinal symptoms in patients with coronavirus disease 19: a systematic review and meta-analysis. Clin Res Hepatol Gastroenterol. (2020) 44:653–61. 10.1016/j.clinre.2020.04.012 32418852 PMC7214284

[18] Zhang C Shi L Wang FS. Liver injury in COVID-19: management and challenges. Lancet Gastroenterol Hepatol. (2020) 5:428–30. 10.1016/S2468-1253(20)30057-1 32145190 PMC7129165

[19] Zhao B Ni C Gao R Wang Y Yang L Wei J. Recapitulation of SARS-CoV-2 infection and cholangiocyte damage with human liver ductal organoids. Protein & cell. (2020) 11:771–5. 10.1007/s13238-020-00718-6 32303993 PMC7164704

[20] Pirola CJ Sookoian S. SARS-CoV-2 virus and liver expression of host receptors: putative mechanisms of liver involvement in COVID-19. Liver Int. (2020) 40:2038–40. 10.1111/liv.14500 32352224 PMC7267350

[21] Sun J Aghemo A Forner A Valenti L. COVID-19 and liver disease. Liver Int. (2020) 40:1278–81. 10.1111/liv.14470 32251539

[22] Guan WJ Ni ZY Hu Y Liang WH Ou CQ He JX. Clinical characteristics of coronavirus disease 2019 in China. N Engl J Med. (2020) 382:1708–20. 10.1056/NEJMoa2002032 32109013 PMC7092819

[23] Kumar MP Mishra S Jha DK Shukla J Choudhury A Mohindra R. Coronavirus disease (COVID-19) and the liver: a comprehensive systematic review and meta-analysis. Hepatol Int. (2020) 14:711–22. 10.1007/s12072-020-10071-9 32623633 PMC7335221

[24] Jothimani D Venugopal R Abedin MF Kaliamoorthy I Rela M. COVID-19 and the liver. J Hepatol. (2020) 73:1231–40. 10.1016/j.jhep.2020.06.006 32553666 PMC7295524

[25] Cai Q Huang D Yu H Zhu Z Xia Z Su Y. COVID-19: abnormal liver function tests. J Hepatol. (2020) 73:566–74. 10.1016/j.jhep.2020.04.006 32298767 PMC7194951

[26] Chai X Hu L Zhang Y Han W Lu Z Ke A. Specific ACE2 expression in cholangiocytes may cause liver damage after 2019-nCoV infection. bioRxiv [preprint]. (2020). 10.1101/2020.02.03.931766

[27] Garrido I Liberal R Macedo G. Review article: COVID-19 and liver disease-what we know on 1st May 2020. Alim Pharmacol Ther. (2020) 52:267–75. 10.1111/apt.15813 32402090 PMC7272838

[28] Tian S Xiong Y Liu H Niu L Guo J Liao M. Pathological study of the 2019 novel coronavirus disease (COVID-19) through postmortem core biopsies. Mod Pathol. (2020) 33:1007–14. 10.1038/s41379-020-0536-x 32291399 PMC7156231

[29] Schurink B Roos E Radonic T Barbe E Bouman CSC de Boer HH. Viral presence and immunopathology in patients with lethal COVID-19: a prospective autopsy cohort study. Lancet Microbe. (2020) 1:e290–9. 10.1016/S2666-5247(20)30144-0 33015653 PMC7518879

[30] Fassan M Mescoli C Sbaraglia M Guzzardo V Russo FP Fabris R. Liver histopathology in COVID-19 patients: a mono-Institutional series of liver biopsies and autopsy specimens. Pathol Res Pract. (2021) 221:153451. 10.1016/j.prp.2021.153451 33932720 PMC8054534

[31] Jarnagin WR Büchler MW Chapman WC D'Angelica MI. Blumgart's Surgery of the Liver, Biliary Tract and Pancreas. Blumgart LH, editor. Philadelphia, PA: Elsevier (2012). p. 1361–91. 10.1016/B978-1-4377-1454-8.00157-0

[32] Zacherl J Scheuba C Imhof M Jakesz R Függer R. Long-term results after laparoscopic unroofing of solitary symptomatic congenital liver cysts. Surg Endosc. (2000) 14:59–62. 10.1007/s004649900012 10653238

